# Supplementary material for: Systematic investigation of recipient cell genetic requirements reveals important surface receptors for conjugative transfer of IncI2 plasmids
Source: Commun Biol. 2023 Nov 16;6:1172. doi: 10.1038/s42003-023-05534-2 (PMC10654706; doi:10.1038/s42003-023-05534-2)
Supplement: Supplementary file 2 — Supplemental Material [file 42003_2023_5534_MOESM2_ESM.pdf]

**Supplemental Materials**

# Systematic investigation of recipient cell genetic requirements reveals important surface receptors for conjugative transfer of IncI2 plasmids

Nancy Allard[1], Arianne Collette[1], Josianne Paquette[1], Sébastien Rodrigue[1]* and Jean-Philippe Côté[1*]

[1] Département de biologie, Faculté des sciences, Université de Sherbrooke, Sherbrooke, QC J1K 2R1, Canada

* Correspondence may be addressed to:

Sébastien Rodrigue
Tel: 1-819-821-8000 ext. 62939
Email : Sebastien.Rodrigue@USherbrooke.ca

Jean-Philippe Côté
Tel :1-819-821-8000 ext. 65280
Email : JP.Cote@USherbrooke.ca

**This Supplemental Material includes:**

Supplementary discussion

Supplementary Tables 1 to 2

Supplementary Figures 1 to 8

Supplementary References

## Supplementary discussion

## LPS structure description

The outer membrane of Gram-negative bacteria is an essential feature acting like a barrier to protect cells from toxic compounds such as antibiotics and detergents[1]. From the outer membrane to the extracellular space, the LPS structure consists of (i) lipid A, a hydrophobic glycolipid which anchors LPS in the outer membrane; (ii) core oligosaccharide (core-OS), a non-repeating oligosaccharide containing sugars such as heptose and keto-deoxyoctulosonate (Kdo); and (iii) O antigen, a polysaccharide made of up to 50 repeating oligosaccharide subunits (Fig. 3 c). LPS biosynthesis involves a large number of enzyme activities, governed by more than 40 genes[2,3] that account for a large structural diversity. In the *Enterobacteriaceae*, the core-OS is divided into two distinct regions, the inner and the outer core-OS (Fig. 3 c). The inner core is highly conserved and comprises three Kdo and L-*glycero*-D-*manno*-heptose (Hep) residues and is often phosphorylated. The outer core-OS comprises a tri-hexose backbone that can be modified with varying side-branch substitutions[4]. *Escherichia coli* serotypes are determined by the type of polysaccharide antigens on their cell membrane including the O antigen. A total of ~178 distinct antigens have been formally defined for *E. coli* alone. This diversity allows bacteria to have a surface that offers a selective advantage in its specific niche[3]. Despite this high degree of diversity in O antigen, only five distinct core-OS structures are found in *E. coli*, designated K-12, R1, R2, R3 and R4 (Fig. 5 b). These core-OS structures are synthesized through the successive addition of various sugars to lipid A by the products of the *waa* genes (formerly *rfa* genes)[4].

## PilV variants recognize receptor structures in the core-OS of *E. coli* LPS

The loss of long O16 antigen in *E. coli* BW25113 (K-12 core-oligosaccharide) is due to the disruption of the rhamnosyl transferase *wbbL* gene with an IS5 element, termed the *rfb-50* mutation[1,5]. Since the complementation of this lesion by insertion of an intact *wbbL* gene from an O antigen-expressing strain of *E. coli* into the

chromosome of *E. coli* BW25113 restored O antigen expression[1,6], all other genes are functional. To ensure that these other genes do not incorporate new sugars into the LPS structure, we generated an *E. coli* BW25113 mutant in which the *rfbABCD*, *wzxB*, *glf* and *wbbHIJKL* genes were removed (Fig. 3 c). This ΔO antigen mutant, exhibiting a core-OS, as well as a mutant displaying only the lipid IVA, called ClearColi[7] (Fig. 3 c), were subjected to pairwise conjugation in broth using all TP114 derivatives bearing a single PilV variant[8] (Fig. 3 d). As previously reported[8], transfer rates of TP114 mutant in which the variable region of *pilV* was replaced by a FLAG tag was virtually unaffected on solid medium, but its capacity to conjugate in broth was severely altered. Moreover, since *E. coli* BW25113 and BW25113ΔO antigen were recognized by the same three adhesins (PilVA', PilVC and PilVC') while ClearColi did not generate any transconjugants, we can infer that these adhesins -and potentially those that did not recognize the K-12 core structure- exhibit specificities for a receptor present in the core-OS.

**Elucidation of receptor structure(s) for PilV variants using knockout mutants**

Results surrounding the elucidation of receptor structures of PilVA' and PilVC are described in detail in the main article and will not be elaborated here. The use of a donor strain expressing only one PilV variant at a time[8] allowed us to study their specificities towards different recipient bacteria. The pairwise mating experiments in broth allowed us to validate receptor structures since strains displaying the receptor structure at the surface of the cell generated transconjugants, while those missing the receptor molecule do not. For example, the PilVA adhesin of plasmid R64 was shown to specifically bind to *N*-acetylglucosamine-β-(1-3)-glucose[9] (Fig. 5 b). This receptor structure is only present in *E. coli* R1 and thus this strain is the only one producing transconjugants when mating with TP114-bearing PilVA adhesin, which is homologous to R64 PilVA. Moreover, it was shown that a Δ*waaL* knockout mutant of *E. coli* R1 was no longer recognized by PilVA adhesin[9]. Consequently, we expect that cloning the genes responsible for the addition of *N*-acetylglucosamine-β-(1-3)-glucose disaccharide and their expression in a strain

representing one of the other core-OS would produce transconjugants when mating with a donor expressing PilVA adhesin.

The conjugation results with the PilVC' variant were also in agreement with the receptor structure proposed for the homolog of R64 as being glucose-α-(1-2)-glucose or glucose-α-(1-2)-galactose. Indeed, in the case of *E. coli* R2 and *E. coli* BW25113 which displays the K-12 core-OS, the glucose-α-(1-2)-glucose is the target of PilVC' adhesin. Effectively, *E. coli* BW25113Δ*waaR*, missing the third glucose, is no longer recognized by PilVC' adhesin (Supp. Fig. 4 b). Moreover, all other knockout mutants affecting the core-OS lower in the structure, such as Δ*waaO* (Supp. Fig. 4 c), Δ*waaG*, Δ*waaF*, Δ*waaC* (Fig. 4 f, g, h) does not generate transconjugants when mating in broth with a donor harbouring the PilVC' adhesin (Fig. 5 a). On the other hand, in *E. coli* R1 and R4, it is the glucose-α-(1-2)-galactose that acts as a receptor structure (Fig. 5). One intriguing thing about those receptors is that both can be found in the outer core-OS of *E. coli* R3, but this strain does not produce any transconjugants when using the PilVC' adhesin. This could mean that the glucose added by WaaG may be important in the receptor structure. To investigate this possibility, the *waaO* and *waaR* genes could be introduced *in trans* in *E. coli* BW25113 Δ*waaG*. If this strain, harbouring a chimeric LPS, is still recognized by the PilVC' adhesin, then the receptor is a disaccharide, but if not, the receptor could be a trisaccharide.

**Elucidation of receptor structure(s) for PilV variants using knock-in mutants**

To explore other potential receptor structures, we cloned one or two *waa* genes originating from another core-OS prototype in an arabinose inducible plasmid (pBAD30). For example, when introducing pWaaOX (bearing *waaO* and *waaX* genes from *E. coli* R4) in *E. coli* R3, this new strain expressing chimeric LPS produced transconjugants when mating in broth with the PilVC' adhesin, which was not the case with the wild type strain (Supp. Fig. 4 d, e). Since *waaO* and *waaX* genes are responsible for the incorporation of the galactose-β-(1-4)-glucose in the LPS, this disaccharide is likely a new receptor structure of PilVC' adhesin.

In our results, only the R2 core LPS and strains in which we added the appropriate genes were recognized by the PilVB variant. More precisely, when introducing *waaK in trans* in *E. coli* BW25113 or *waaK* along with *waaR* genes in *E. coli* R4 (Supp. Fig. 4 f, g, h), those strains produced transconjugants when mating in broth with a donor strain harbouring PilVB adhesin, while the wild type strains did not (Supp. Fig. 7). Since *waaK* is responsible for the addition of an *N*-acetylglucosamine sugar on a glucose molecule with an α-(1-2) linker, this modification could occur on the first glucose of BW25113 LPS, thus allowing transconjugants to form upon mating assays in broth using the PilVB adhesin (Fig. 4). This again highlights the fact that the nature of the linker between the two molecules is crucial since there is already an *N*-acetylglucosamine-α-(1-6)-glucose in the O16 antigen of *E. coli* BW25113[5], but the PilVB adhesin does not recognize the wild-type strain. *N*-acetylglucosamine-α-(1-2)-glucose structure was previously proposed as the target of PilVB' for R64[10]. However, our results show that *N*-acetylglucosamine-α-(1-2)-glucose is rather the receptor structure for the PilVB variant.

The introduction of pWaaD, pWaaT, pWaaV, pWaaW, pWaaJ, pWaaVL, pWaaRK, pWaaTW-R1 or pWaaTW-R4 into *E. coli* BW25113 and expression of chimeric LPS did not change the pattern of PilV adhesin recognition of the strains (Supp. Fig. 7).

**Supplementary Table 1. Strains and plasmids used in this study.**

| Strain or plasmid | Description[a] | Source (reference) |
|---|---|---|
| ***Escherichia coli*** | | |
| EC100D*pir+* | F- *mcrA* Δ(*mrr-hsdRMS-mcrBC*) ϕ80*dlacZ*Δ*M15* Δ*lacX74 recA1 endA1 araD139* Δ(*ara, leu*)*7697 galU galK* λ– *rpsL nupG pir*+ (DHFR) | #ECP09500 (Lucigen) |
| Nissle 1917 | Wild type probiotic strain | Brady *et al.* 2013 [11] |
| KN01Δ*dapA* | Nissle 1917Δ*dapA*, $Sp^R$, $Sm^R$ | Neil *et al.* 2020 [12] |
| VB111 | $MG1655Nx^R$ , K-12 F- λ- *ilvG*– *rfb-50 rph-1* | Ceccarelli *et al.* 2008 [13] |
| BW25113 | F- Δ(*araD-araB*)*567*, Δ*lacZ4787*(::*rrnB-3*), λ-, *rph-1*, Δ(*rhaD-rhaB*)*568*, *hsdR514* | CGSC #7636 |
| BW25113ΔO antigen::$Kn^R$ | BW25113 where O antigen (*rfbABCD*, *wzxB*, *glf*, *wbbHIJKL*) have been replace by $Kn^R$ by recombineering | This study |
| BW25113Δ*waaB*::$Kn^R$ | Keio mutant JW3603 | Baba *et al.* 2006 [14] |
| BW25113Δ*waaC*::$Kn^R$ | Keio mutant JW3596 | Baba *et al.* 2006 [14] |
| BW25113Δ*waaE*::$Kn^R$ | Keio mutant JW3024 | Baba *et al.* 2006 [14] |
| BW25113Δ*waaF*::$Kn^R$ | Keio mutant JW3595 | Baba *et al.* 2006 [14] |
| BW25113Δ*waaG*::$Kn^R$ | Keio mutant JW3606 | Baba *et al.* 2006 [14] |
| BW25113Δ*waaL*::$Kn^R$ | Keio mutant JW3597 | Baba *et al.* 2006 [14] |
| BW25113Δ*waaO*::$Kn^R$ | Keio mutant JW3602 | Baba *et al.* 2006 [14] |
| BW25113Δ*waaP*::$Kn^R$ | Keio mutant JW3605 | Baba *et al.* 2006 [14] |
| BW25113Δ*waaQ*::$Kn^R$ | Keio mutant JW3607 | Baba *et al.* 2006 [14] |
| BW25113Δ*waaR*::$Kn^R$ | Keio mutant JW3601 | Baba *et al.* 2006 [14] |
| BW25113Δ*waaS*::$Kn^R$ | Keio mutant JW3604 | Baba *et al.* 2006 [14] |
| BW25113Δ*waaY*::$Kn^R$ | Keio mutant JW3600 | Baba *et al.* 2006 [14] |
| BW25113Δ*waaZ*::$Kn^R$ | Keio mutant JW3599 | Baba *et al.* 2006 [14] |
| BW25113Δ*gmhB*::$Kn^R$ | Keio mutant JW0196 | Baba *et al.* 2006 [14] |
| BW25113Δ*gmhD*::$Kn^R$ | Keio mutant JW3594 | Baba *et al.* 2006 [14] |

**Supplementary Table 1. Strains and plasmids used in this study (continued).**

| Strain or plasmid | Description[a] | Source (reference) |
|---|---|---|
| BW25113Δ*hlyT*::$Kn^R$ | Keio mutant JW3818 | Baba *et al.* 2006 [14] |
| BW25113Δ*kdsC*::$Kn^R$ | Keio mutant JW3165 | Baba *et al.* 2006 [14] |
| F470† | Standard for R1 prototype O-:K-, R-LPS mutant of O8:K27 | Tsang *et al.* 1987 [15] |
| F632† | Standard for R2 prototype O-:K-, R-LPS mutant of O100 | Tsang *et al.* 1987 [15], Hämmerling *et al.* 1971 [16] |
| F653† | Standard for R3 prototype O-:K-, R-LPS mutant of O111:K58 | Tsang *et al.* 1987 [15] |
| F2513† | Standard for R4 prototype O-:K-, R-LPS mutant of O14:K7 | Tsang *et al.* 1987 [15] |
| ClearColi | BL21(DE3) *msbA148* Δ*gutQ* Δ*kdsD* Δ*lpxLMP* Δ*pagP* Δ*eptA* | Mamat *et al.* 2013 [7] |
| **Plasmid** | | |
| pBAD30 | $oriV_{p15A}$, *bla* ($Ap^R$), *araC*, $P_{BAD}$ | Guzman *et al.* 1995 [17] |
| pE-FLP | $oriV_{pSC101ts}$, *flp*, *bla* ($Ap^R$) | Addgene #45978 |
| pKD3 | $oriV_{R6K}$, FRT flanked $Cm^R$, $Ap^R$, template for one-step recombineering | Addgene #45604 |
| pKD4 | $oriV_{R6K}$, FRT flanked $Kn^R$, $Ap^R$, template for one-step recombineering | Addgene #45605 |
| pSIM6 | $oriV_{pSC101ts}$, Lambda Red recombinase, *bla* ($Ap^R$) | Datta *et al.*, 2006 [18] |
| pWaaD | pBAD30, *waaD* under the control of $P_{BAD}$ | Addgene #210448 |
| pWaaID | pBAD30, *waaI* and *waaD* under the control of $P_{BAD}$ | Addgene #210449 |
| pWaaJ | pBAD30, *waaJ* under the control of $P_{BAD}$ | Addgene #210450 |
| pWaaK | pBAD30, *waaK* under the control of $P_{BAD}$ | Addgene #210451 |
| pWaaRK | pBAD30, *waaR* and *waaK* under the control of $P_{BAD}$ | Addgene #210452 |
| pWaaT | pBAD30, *waaT* under the control of $P_{BAD}$ | Addgene #210453 |
| pWaaW | pBAD30, *waaW* under the control of $P_{BAD}$ | Addgene #210454 |

**Supplementary Table 1. Strains and plasmids used in this study (continued).**

| Strain or plasmid | Description[a] | Source (reference) |
|---|---|---|
| pWaaTW-R1 | pBAD30, *waaT* and *waaW* from R1 under the control of $P_{BAD}$ | Addgene #210455 |
| pWaaTW-R4 | pBAD30, *waaT* and *waaW* from R4 under the control of $P_{BAD}$ | Addgene #210456 |
| pWaaV | pBAD30, *waaV* under the control of $P_{BAD}$ | Addgene #210457 |
| pWaaVL | pBAD30, *waaV* and *waaL* under the control of $P_{BAD}$ | Addgene #210458 |
| pWaaX | pBAD30, *waaX* under the control of $P_{BAD}$ | Addgene #210459 |
| pWaaOX | pBAD30, *waaO* and *waaX* under the control of $P_{BAD}$ | This study |
| TP114 | IncI2 conjugative plasmid, *aph*(3')-1 ($Kn^R$) | DSM-4246 (DSMZ) |
| TP114ΔKn::Cm | TP114 deletion mutant for the Kn resistance gene, replaced by the Cm resistance gene | This study |
| TP114Δ*pilV*::FLAG-*cat* | TP114 deletion mutant for the variable 3'-end of *pilV* gene replaced by a FLAG-tag, FRT flanked $Cm^R$ | Allard *et al.* 2022 [8] |
| TP114Δshufflon::*pilVA-cat* | TP114 deletion mutant for the shufflon with *pilVA* fixed | Allard *et al.* 2022 [8] |
| TP114Δshufflon::*pilVA'-cat* | TP114 deletion mutant for the shufflon with *pilVA'* fixed | Allard *et al.* 2022 [8] |
| TP114Δshufflon::*pilVB-cat* | TP114 deletion mutant for the shufflon with *pilVB* fixed | Allard *et al.* 2022 [8] |
| TP114Δshufflon::*pilVB'-cat* | TP114 deletion mutant for the shufflon with *pilVB'* fixed | Allard *et al.* 2022 [8] |
| TP114Δshufflon::*pilVC-cat* | TP114 deletion mutant for the shufflon with *pilVC* fixed | Allard *et al.* 2022 [8] |
| TP114Δshufflon::*pilVC'-cat* | TP114 deletion mutant for the shufflon with *pilVC'* fixed | Allard *et al.* 2022 [8] |
| TP114Δshufflon::*pilVD-cat* | TP114 deletion mutant for the shufflon with *pilVD* fixed | Allard *et al.* 2022 [8] |
| TP114Δshufflon::*pilVD'-cat* | TP114 deletion mutant for the shufflon with *pilVD'* fixed | Allard *et al.* 2022 [8] |
| TP114Δshufflon::*pilVA-R64-cat* | TP114 deletion mutant for the shufflon with R64 *pilVA* fixed | This study |
| TP114Δshufflon::*pilVA'-R64-cat* | TP114 deletion mutant for the shufflon with R64 *pilVA'* fixed | This study |
| TP114Δshufflon::*pilVB-R64-cat* | TP114 deletion mutant for the shufflon with R64 *pilVB* fixed | This study |

**Supplementary Table 1. Strains and plasmids used in this study (continued).**

| Strain or plasmid | Description[a] | Source (reference) |
|---|---|---|
| TP114Δshufflon::*pilVB'-R64-cat* | TP114 deletion mutant for the shufflon with R64 *pilVB'* fixed | This study |
| TP114Δshufflon::*pilVC-R64-cat* | TP114 deletion mutant for the shufflon with R64 *pilVC* fixed | This study |
| TP114Δshufflon::*pilVC'-R64-cat* | TP114 deletion mutant for the shufflon with R64 *pilVC'* fixed | This study |
| TP114Δshufflon::*pilVD'-R64-cat* | TP114 deletion mutant for the shufflon with R64 *pilVD'* fixed | This study |

[a] $Ap^R$, ampicillin; $Cm^R$, chloramphenicol; $Kn^R$, kanamycin; $Nx^R$, nalidixic acid; $Sp^R$, spectinomycin; DAP, diaminopimelic acid; ts, thermosensitive.

† Gift from Chris Whitfield, original from Max-Planck-Institute collection.

**Supplementary Table 2: Oligonucleotides used in this study.**

| Context | Name | Sequence[a, b] (5'-3') | Template | Amplicon |
|---|---|---|---|---|
| BW25113ΔO antigen::Kn$^R$ | oKn-F | atacctctattaatcaaactgagagccgcttatttcacagagattgcagcattacacgtc | pKD4 | FRT flanked Kn$^R$ |
| | oKn-R | ataaatagcttatccatgcttaaatgcttaacggctttatatggtccatatgaatatcctc | | |
| | oBW1-F | gctcgtcacatcataggc | BW25113ΔO antigen | Screen O antigen deletion clones |
| | oBW1-R | cggcctggaatgttcgca | | |
| TP114ΔKn::Cm | aph-III-pKD3-F | gtgcccgccgatgcgccaaccgcattcattaaagactaactagtaggctggagctgcttc | pKD3 | FRT flanked Cm$^R$ |
| | aph-III-pKD3-R | ataaaactgtctgcttacataaacagtaatacaaggggtgttgggaattagccatggtcc | | |
| TP114 *pilV* variants derivatives | ocat-F | atgggaattagccatggtcc | pKD3 | FRT flanked Cm$^R$ |
| | opilV1-R | taaaaaatcactctgtagttgttctatcataagtgaatccgtgtaggctggagctgcttc | | |
| TP114Δshufflon::*pilVA-R64-cat* | opilV22-F | cagtacaggggcgatactttcgtgccaatccggtcggtggaagacttctggttcgctcaa | R64 | *pilVA* |
| | opilV22-R | ggaccatggctaattcccatttaagtttgatatccaaatactg | | |
| TP114Δshufflon::*pilVA'-R64-cat* | opilV23-F | cagtacaggggcgatactttcgtgccaatccggtacgtgggggacaataggtggaaaact | R64 | *pilVA'* |
| | opilV23-R | ggaccatggctaattcccatttaattgagcgttacacacgacgc | | |
| TP114Δshufflon::*pilVB-R64-cat* | opilV24-F | cagtacaggggcgatactttcgtgccaatccggtacctggaaatcatcatcagcgtcgat | R64 | *pilVB* |
| | opilV24-R | ggaccatggctaattcccatctatttcaagcaagtaatcttcataaagtagga | | |
| TP114Δshufflon::*pilVB'-R64-cat* | opilV25-F | cagtacaggggcgatactttcgtgccaatccggtacctggagaaaggtaggatctggtga | R64 | *pilVB'* |
| | opilV25-R | ggaccatggctaattcccatttattggcaaatggcatatacagttattgaac | | |
| TP114Δshufflon::*pilVC-R64-cat* | opilV26-F | cagtacaggggcgatactttcgtgccaatccggtacctggggcgctcctaaaattcaatt | R64 | *pilVC* |
| | opilV26-R | ggaccatggctaattcccatttaagctccagcaccaggaaga | | |
| TP114Δshufflon::*pilVC'-R64-cat* | opilV27-F | cagtacaggggcgatactttcgtgccaatccggtcggtggtcaggggggtaataaaattaa | R64 | *pilVC'* |
| | opilV27-R | ggaccatggctaattcccatttaattaaaggggcaacagtaggca | | |
| TP114Δshufflon::*pilVD'-R64-cat* | opilV28-F | cagtacaggggcgatactttcgtgccaatccggtacttggcgtaaaagcaattctggtag | R64 | *pilVD'* |
| | opilV28-R | ggaccatggctaattcccattcagttctgacacgcgacga | | |
| pWaa plasmid series | opBAD30-F | gcatgcaagcttggctgttttg | pBAD30 | pBAD30 backbone |
| | opBAD30-R | ttttgcctcctagagctcgaattcgctagccc | | |

**Supplementary Table 2: Oligonucleotides used in this study (continued).**

| Context | Name | Sequence[a, b] (5'-3') | Template | Amplicon |
|---|---|---|---|---|
| pWaaD | owaa1-F | tcgagctctaggaggcaaaaatggttgataaaataatatttacggtt | F653 | *waaD* |
| | owaa1-R | aaacagccaagcttgcatgcttaaacaaaccaattatgaataacct | | |
| pWaaID | owaa2-F | tcgagctctaggaggcaaaaatgtctcaactcaatgatag | F653 | *waaI* |
| | owaa2-R | accttaaatctttagaagcatt | | |
| | owaa3-F | tgcttctaaagatttaaggttatacgagatggttgataaa | F653 | *waaD* |
| | owaa1-R | aaacagccaagcttgcatgcttaaacaaaccaattatgaataacct | | |
| pWaaJ | owaa4-F | tcgagctctaggaggcaaaaatgaaattggattttaaacatcttact | F653 | *waaJ* |
| | owaa4-R | aaacagccaagcttgcatgcttaacctttcataacattatatttaattgc | | |
| pWaaK | owaa5-F | tcgagctctaggaggcaaaaatgattaaaaaaataatatttaccgtca | F632 | *waaK* |
| | owaa5-R | aaacagccaagcttgcatgcttaaccaaaccagtcttgtatt | | |
| pWaaRK | owaa6-R | tcgagctctaggaggcaaaagtgaactcatttcctgccat | F632 | *waaR* |
| | owaa6-F | gttaaaccagttaaatgttatttacg | | |
| | owaa7-F | taacatttaactggtttaacgagttggctaataatgattaa | F632 | *waaK* |
| | owaa5-R | aaacagccaagcttgcatgcttaaccaaaccagtcttgtatt | | |
| pWaaRK | owaa6-R | tcgagctctaggaggcaaaagtgaactcatttcctgccat | F632 | *waaR* |
| | owaa6-F | gttaaaccagttaaatgttatttacg | | |
| | owaa7-F | taacatttaactggtttaacgagttggctaataatgattaa | F632 | *waaK* |
| | owaa5-R | aaacagccaagcttgcatgcttaaccaaaccagtcttgtatt | | |
| pWaaT | owaa8-F | tcgagctctaggaggcaaaaatgaatgaatttataaaagaacggttttcg | Nissle 1917 | *waaT* |
| | owaa8-R | aaacagccaagcttgcatgcttatttcttaagcttgtacttaattaatgaa | | |
| pWaaW | owaa9-F | tcgagctctaggaggcaaaaatggatttattagctgagagtatt | Nissle 1917 | *waaW* |
| | owaa9-R | aaacagccaagcttgcatgcttattgcccaaatagttttctttta | | |
| pWaaTW-R1 | owaa8-F | tcgagctctaggaggcaaaaatgaatgaatttataaaagaacggttttcg | Nissle 1917 | *waaT* |
| | owaa7-R | aagagtaatttatttcttaagcttgt | | |
| | owaa10-F | acaagcttaagaaataaattactcttatggatttattagctgagag | Nissle 1917 | *waaW* |
| | owaa9-R | aaacagccaagcttgcatgcttattgcccaaatagttttctttta | | |
| pWaaV | owaa11-F | tcgagctctaggaggcaaaaatgagcaatgattaccctttt | Nissle 1917 | *waaV* |
| | owaa11-R | aaacagccaagcttgcatgccctaacgtttattctttttctatttacc | | |
| pWaaVL | owaa11-F | tcgagctctaggaggcaaaaatgagcaatgattaccctttt | Nissle 1917 | *waaVL* |
| | owaa10-R | aaacagccaagcttgcatgcttacttatctaataaacattgg | | |

**Supplementary Table 2: Oligonucleotides used in this study (continued).**

| Context | Name | Sequence[a, b] (5'-3') | Template | Amplicon |
|---|---|---|---|---|
| pWaaX | owaa12-F | tcgagctctaggaggcaaaaatgaacttgcccatttatattgt | F2513 | *waaX* |
| | owaa12-R | aaacagccaagcttgcatgctcatttcaaaagagaaaaaaaagcc | | |
| pWaaOX | owaa13-F | tcgagctctaggaggcaaaaatgagtgcccactattttaa | F2513 | *waaO* |
| | owaa13-R | taaatgggcaagttcattaattaacttttctcaatttcattt | | |
| | owaa14-F | aaatgaaattgagaaaagttaattaatgaacttgcccattta | F2513 | *waaX* |
| | owaa12-R | aaacagccaagcttgcatgctcatttcaaaagagaaaaaaaagcc | | |
| pWaaTW-R4 | owaa8-F | tcgagctctaggaggcaaaaatgaatgaatttataaaagaacggttttcg | F2513 | *waaT* |
| | owaa14-R | ttccgttttgactgtgtggtttatttcttaagcttgtacttaatt | | |
| | owaa15-F | agtacaagcttaagaaataaaccacacagtcaaaacggaa | F2513 | *waaW* |
| | owaa15-R | aaacagccaagcttgcatgcatctctgggtgattaatcttaataatta | | |

[a]Underline represent binding nucleotides

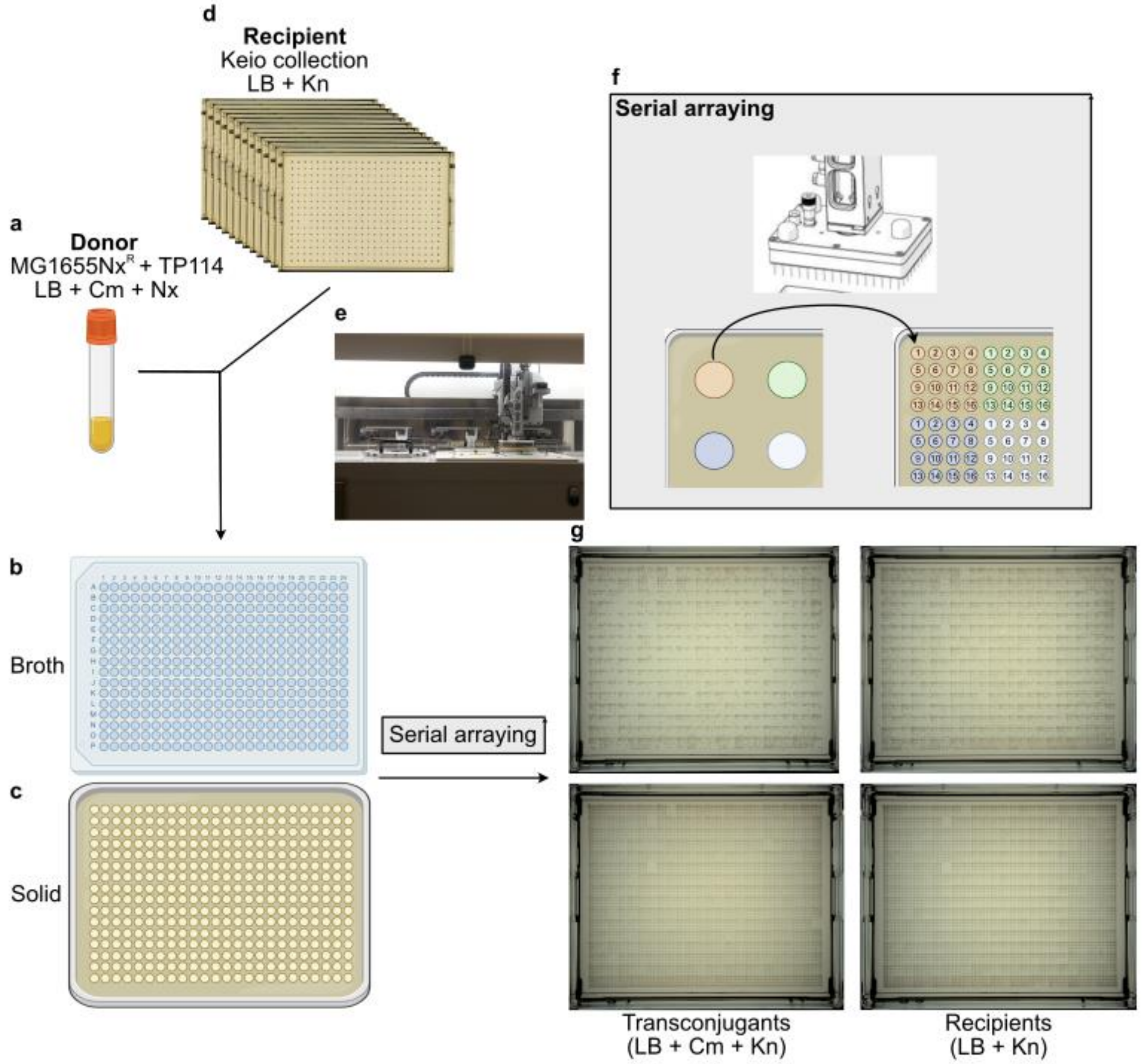


**Supplementary Figure 1 | High throughput conjugation assays.**

Our protocol allows the transfer of a conjugative plasmid from a donor strain towards the 3985 single-gene deletion mutants from the Keio collection, and the 141 small RNA/small protein deletion mutants. The determination of a conjugation score is then calculated for every possible mutant. **a**, The donor bacterium (*E. coli* MG1655Nx$^R$ + TP114) is grown overnight at 37°C in LB containing chloramphenicol (selective marker of TP114) and nalidixic acid, normalized to an OD600nm of 0.1, and transferred into 384-well mating plates (**b**, broth mating) or onto LB-agar mating plates (**c**, solid mating). **d**, At the same time, the deletion mutants are grown overnight at 37°C in LB with kanamycin (selection marker of the mutants) in 384-well plates and transferred to the 384-well and LB-agar mating

plates using the Singer Rotor HDA (**e**). **f**, After 6h of conjugation at 37°C, mating plates were replicated using the Singer Rotor HDA on two selection plates, containing kanamycin and chloramphenicol to select the transconjugants (deletion mutants that have acquired TP114) or kanamycin only to select recipient bacteria. To do so, each conjugation was replicated 16 times sequentially to create a serial dilution array to be able to assess the transfer rate for each mutant. Plates are then incubated at 37°C overnight before being imaged (**g**), and the growth of each mutant is converted into a density value. Finally, a ratio between transconjugant density and recipient density is made to determine the conjugation score of each mutant.

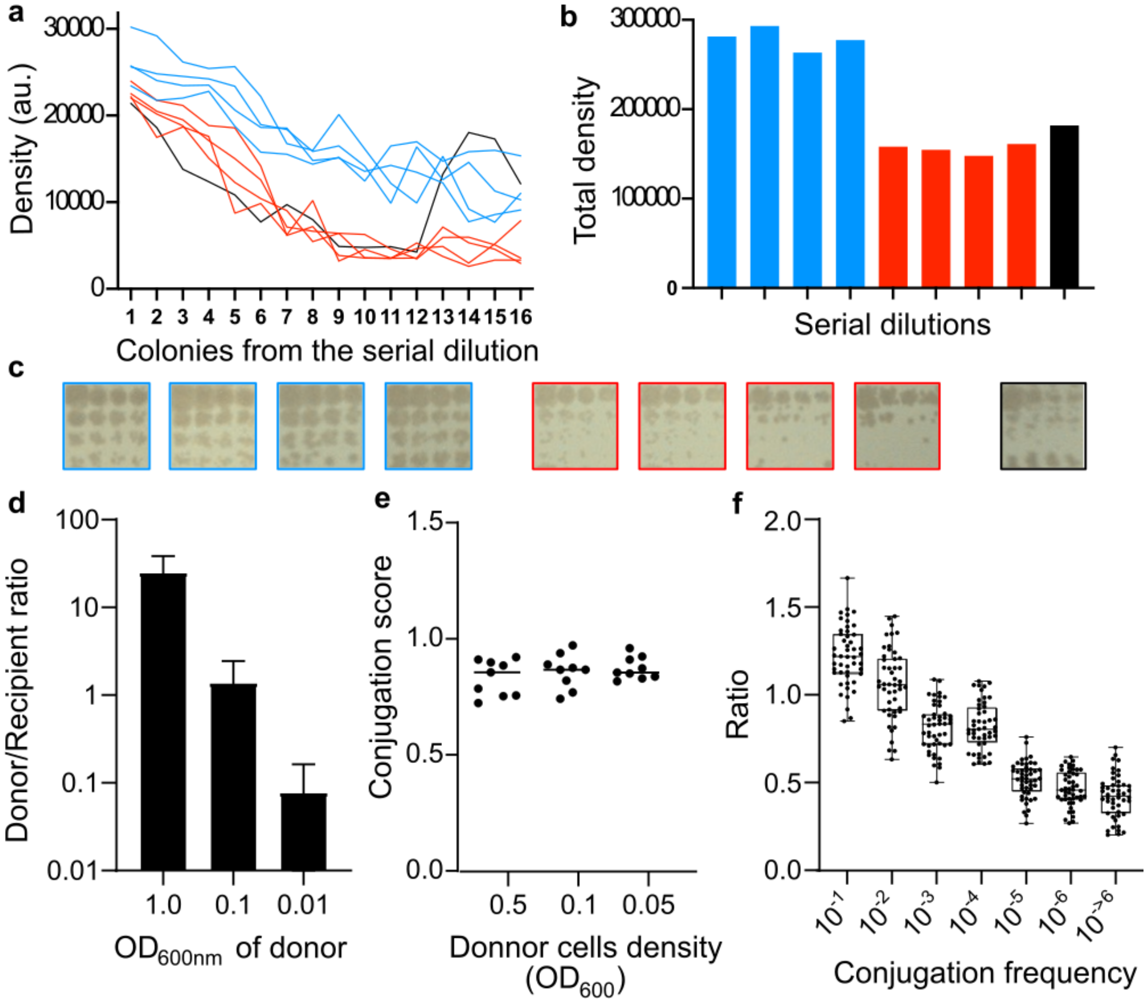


**Supplementary Figure 2 | High-throughput screen validation.** To determine the linear range of the serial dilution from our high-throughput assay, we randomly selected four high-density wells (blue), four low-density wells (red) and one well with colonies at later dilution spots from a recipient plate of the screen (black) for liquid mating. We then measured the density of individual dilution spots in FIJI. (**a**) Density of the 16 individual dilution spots from the 9 wells. (**b**) Total density of the 9 wells. (**c**) Image of the selected wells. (**d**) To measure the ratio of donor to recipient cells in high-throughput conjugation assays, the donor cells harboring conjugative plasmid TP114 were resuspended at an $OD_{600nm}$ of 1.0, 0.1 or 0.01, and 50 μL of these suspensions were added to a 384-well plate. The recipient strain *E. coli* BW25113Δ*yejO* – a deletion mutant of a pseudogene in *E. coli* K-12, was then pinned into the 384-well plates. CFUs were then counted directly from these wells (at T=0 of conjugation). Each bar shows the average ratio for 8 wells. (**e**) Donor cells harboring TP114 were resuspended at various $OD_{600nm}$ and mixed

with the *E. coli* BW25113Δ*yejO* recipient strain to vary donor to recipient ratios. After a 6h conjugation time, transconjugants (Kn and Cm) and recipients (Kn) were selected and the transfer rate was measured as described in the manuscript. (**f**) TP114 variants with different transfer rates were used in the standard manual conjugation assay or high-throughput screening approach. The TP114 variants were hosted in *E. coli* MG1655$Nx^R$ as the donor and were transferred to the *E. coli* BW25113Δ*yejO* recipient strain in broth using the high-throughput conjugation assay. The results suggest that conjugation scores track relatively well with manual transfer rates with a detection limit around $10^{-5}$.

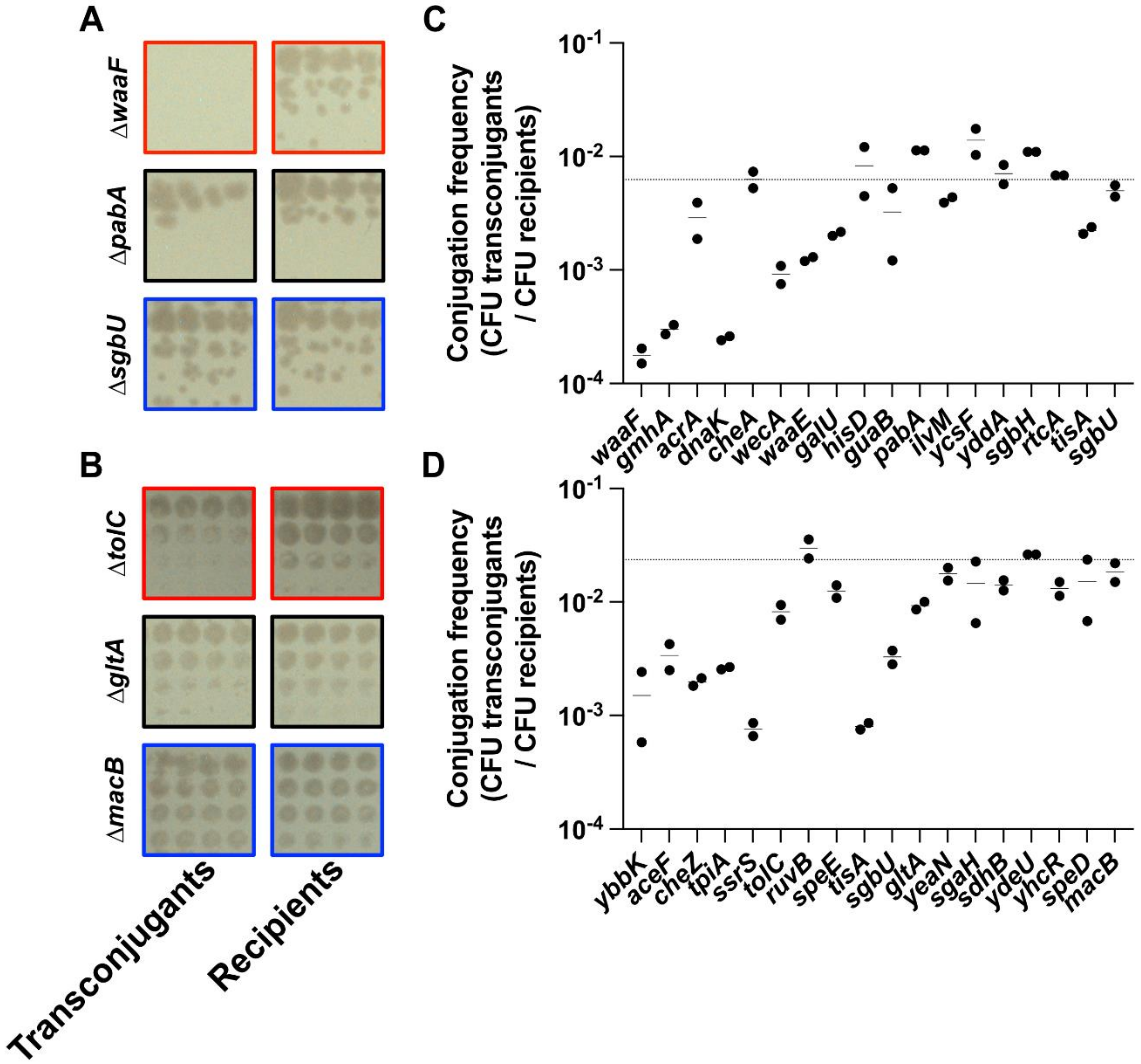


**Supplementary Figure 3 | Transconjugants and recipients growth after high-throughput conjugation assays.**

Examples of the serial dilution growth after high-throughput conjugation assays in broth (**a**) and on a solid support (**b**). Each square represents the sequential replicate (16 in total) to create a serial dilution array and allow us to assess the transfer rate of each mutant. Transconjugants grew on LB plates containing Kn (selection marker of the Keio collection) and Cm (selection marker of the conjugative plasmid TP114), whereas recipients grew on LB plates containing Kn only. Deletion mutants were selected according to their low, medium, and high conjugation scores. Conjugation efficiency of selected genes in broth (**c**) or on solid (**d**) using a standard manual conjugation assay to validate the results of the screen.

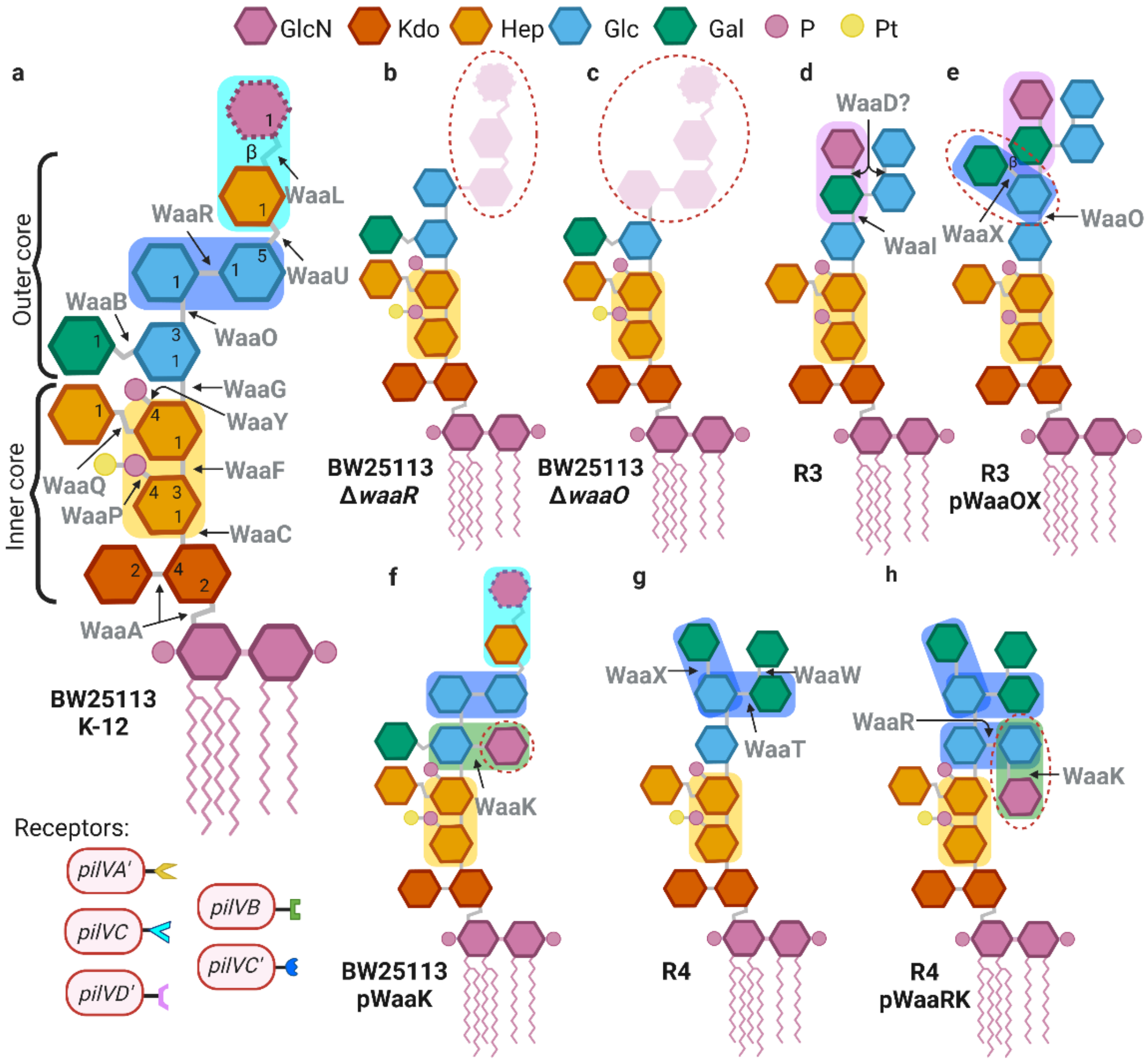


**Supplementary Figure 4 | Comparison of LPS from *E. coli* strains. a**, The LPS structure of *E. coli* BW25113 with a K-12 core-OS is shown. The genes whose products catalyze the formation of each linkage are shown in gray. **b-h**, The expected LPS structures of some knockout[52] and knock-in mutants used in this study. The residues that are lost are depicted in pink and they are circled in red dotted lines. The LPS structure of *E. coli* R4 is also shown (**g**) and the expected LPS structure of *E. coli* R4 transformed with pWaaRK (**h**). All glycoses are in the *α*-anomeric configuration unless stated otherwise. The specific receptor structures for the different PilV adhesins in the LPS molecules are indicated in colored rectangles. Abbreviations of monosaccharide residues: GlcN, glucosamine; Kdo,

2-keto-3-deoxyoctulosonic acid (3-deoxy-D-*manno*-octulosonic acid); Hep, L-*glycero*-D-*manno*-heptose; Glc, glucose; Gal, galactose; P, phosphate; Pt, pyrophosphoethanolamine. Created in BioRender.com.

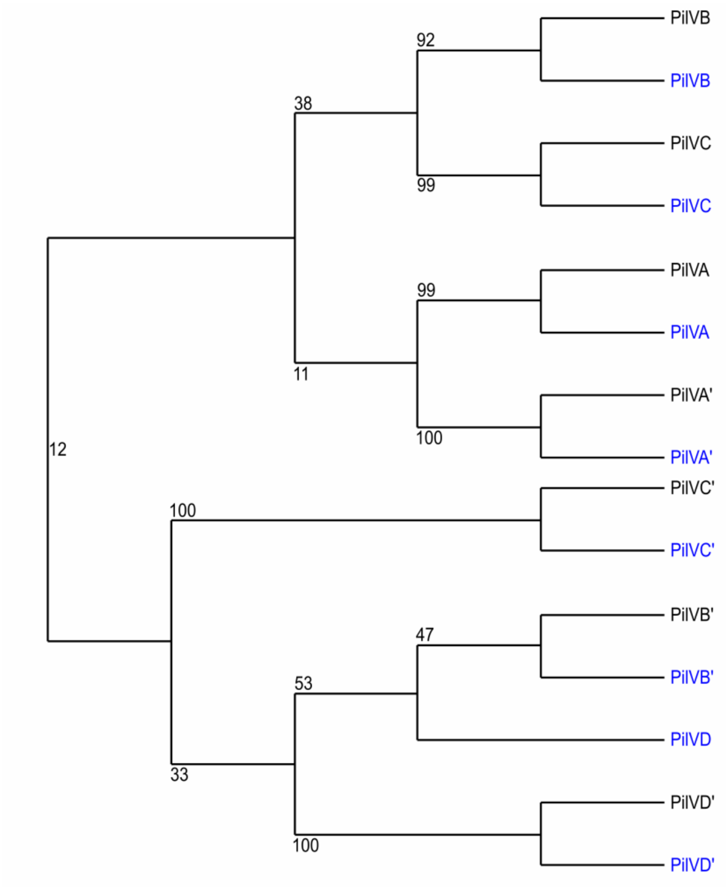


**Supplementary Figure 5 | Phylogenetic tree of PilV adhesins from conjugative plasmids R64 and TP114.**

Phylogenetic tree showing the relationship among the variable C-terminal minor adhesins (PilV) amino acid sequences of conjugative plasmids R64 (AP005147.1) and TP114 (MF521836.2). All analyzed C-terminal adhesins from R64 are paired with their homologue in TP114. The PilVD variant from TP114 seems to have probably evolved from PilVB’ adhesin. The tree was generated by maximum likelihood analysis in SeaView 5.0.4 software. Bootstrap analysis was performed on 1000 trees and the values are provided on the branch nodes. TP114 adhesins are indicated in blue.

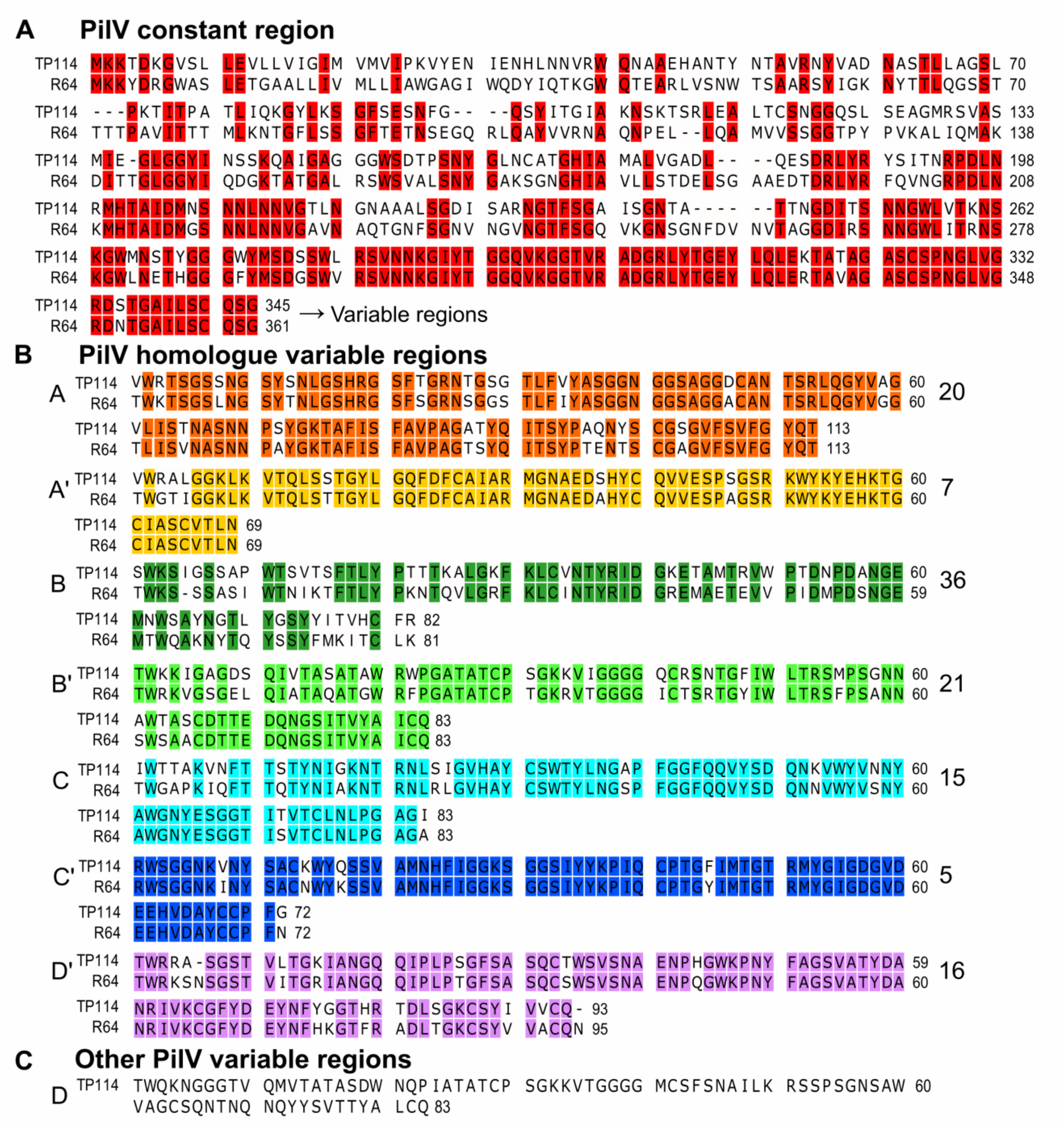

**Supplementary Figure 6 | Sequence comparison of PilV adhesins from conjugative plasmids R64 and TP114.**

Amino acid sequences of constant and variable regions from adhesins displayed by conjugative plasmids R64 (AP005147.1) and TP114 (MF521836.2). Sequence alignments were performed with CLC Main Workbench. The amino acid residues conserved between each homologue are indicated in color. The number of mismatched amino acids is indicated on the right of the alignments.

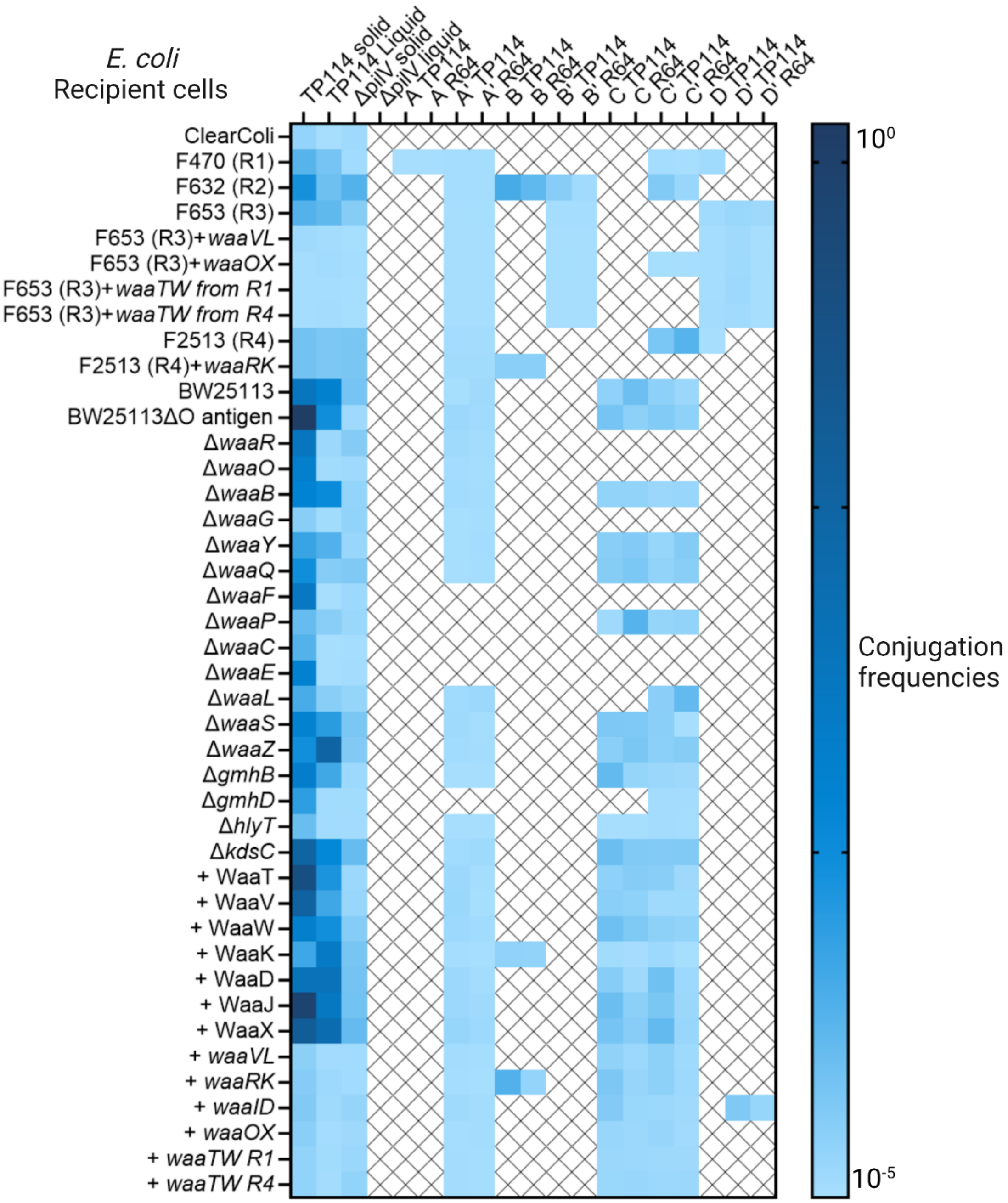


**Supplementary Figure 7 | Conjugation transfer rates of TP114 derivatives.**

Heat map illustrating the transfer rates of wild-type TP114 along with all the derived plasmids expressing only one variant of PilV either from TP114 or R64 against various *E. coli* recipient cells. All conjugations were performed in biological triplicate with *E. coli* Nissle 1917Δ*dapA* as the donor strain (n=3). Cross marks indicate conjugation frequencies below the detection limit of the experiment (1 x $10^{-8}$) in most cases. However, to avoid false positive results, we considered that

the conjugation results underneath $5.9 \times 10^{-5}$ were not significant and were identified with a cross mark as well.

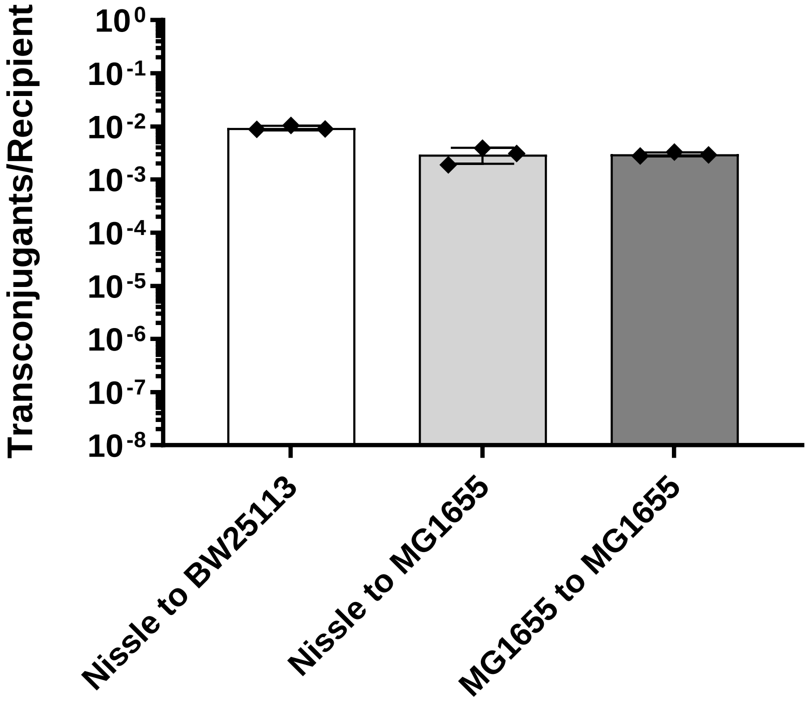


**Supplementary Figure 8 | Impact of different donor and recipient strain combinations on the transfer of conjugative plasmid TP114.**

Three different combinations of donor and recipient strains were assayed in LB broth for 2-hour at 37ºC before plating on a appropriate selective medium and counting the number of CFU to obtain transfer rates (n=3).

**Supplementary References.**


1. Browning, D. F. *et al.* Laboratory adapted *Escherichia coli* K-12 becomes a pathogen of *Caenorhabditis elegans* upon restoration of O antigen biosynthesis. *Mol. Microbiol.* **87**, 939–950 (2013).
2. Whitfield, C. & Valvano, M. A. Biosynthesis and expression of cell-surface polysaccharides in Gram-negative bacteria. *Adv. Microb. Physiol.* **35**, 135–246 (1993).
3. Liu, B. *et al.* Structure and genetics of *Escherichia coli* O antigens. *FEMS Microbiol. Rev.* **44**, 655–683 (2020).
4. Heinrichs, D. E., Yethon, J. A. & Whitfield, C. Molecular basis for structural diversity in the core regions of the lipopolysaccharides of *Escherichia coli* and *Salmonella enterica*. *Mol. Microbiol.* **30**, 221–232 (1998).
5. Feldman, M. F. *et al.* The activity of a putative polyisoprenol-linked sugar translocase (Wzx) involved in *Escherichia coli* O antigen assembly is independent of the chemical structure of the O repeat. *J. Biol. Chem.* **274**, 35129–35138 (1999).
6. Stevenson, G. *et al.* Structure of the O antigen of *Escherichia coli* K-12 and the sequence of its *rfb* gene cluster. *J. Bacteriol.* **176**, 4144–4156 (1994).
7. Mamat, U. *et al.* Endotoxin-free protein production—ClearColi$^{TM}$ technology. *Nat. Methods* **10**, 916 (2013).
8. Allard, N., Neil, K., Grenier, F. & Rodrigue, S. The type IV pilus of plasmid TP114 displays adhesins conferring conjugation specificity and is important for DNA transfer in the mouse gut microbiota. *Microbiol. Spectr.* **10**, e0230321 (2022).
9. Ishiwa, A. & Komano, T. PilV adhesins of plasmid R64 thin pili specifically bind to the lipopolysaccharides of recipient cells. *J. Mol. Biol.* **343**, 615–625 (2004).
10. Ishiwa, A. & Komano, T. Thin pilus PilV adhesins of plasmid R64 recognize specific structures of the lipopolysaccharide molecules of recipient cells. *J. Bacteriol.* **185**, 5192–5199 (2003).
11. Cress, B. F., Linhardt, R. J. & Koffas, M. A. G. Draft genome sequence of *Escherichia coli* strain Nissle 1917 (serovar O6:K5:H1). *Genome Announc.* **1**, e0004713 (2013).
12. Neil, K., Allard, N., Grenier, F., Burrus, V. & Rodrigue, S. Highly efficient gene transfer in the mouse gut microbiota is enabled by the Incl2 conjugative plasmid TP114. *Commun. Biol.* **3**, 523 (2020).
13. Ceccarelli, D., Daccord, A., René, M. & Burrus, V. Identification of the origin of transfer (*oriT*) and a new gene required for mobilization of the SXT/R391 family of integrating conjugative elements. *J. Bacteriol.* **190**, 5328–5338 (2008).
14. Baba, T. *et al.* Construction of *Escherichia coli* K-12 in-frame, single-gene knockout mutants: The Keio collection. *Mol. Syst. Biol.* **2**, 2006.0008 (2006).
15. Tsang, R. S. *et al.* A murine monoclonal antibody specific for the outer core oligosaccharide of *Salmonella* lipopolysaccharide. *Infect. Immun.* **55**, 211–

216 (1987).
16. Hämmerling, G., Lüderitz, O., Westphal, O. & Mäkelä, P. H. Structural investigations on the core polysaccharide of *Escherichia coli* O100. *Eur J Biochem* **22**, 331–344 (1971).
17. Guzman, L. M., Belin, D., Carson, M. J. & Beckwith, J. Tight regulation, modulation, and high-level expression by vectors containing the arabinose PBAD promoter. *J. Bacteriol.* **177**, 4121–4130 (1995).
18. Datta, S., Costantino, N. & Court, D. L. A set of recombineering plasmids for Gram-negative bacteria. *Gene.* **379**, 109–115 (2006).
19. Nakao, R., Ramstedt, M., Wai, S. N. & Uhlin, B. E. Enhanced biofilm formation by *Escherichia coli* LPS mutants defective in Hep biosynthesis. *PLoS One* **7**, e51241 (2012).
