# Supplementary material for: Systematic investigation of recipient cell genetic requirements reveals important surface receptors for conjugative transfer of IncI2 plasmids
Source: Commun Biol. 2023 Nov 16;6:1172. doi: 10.1038/s42003-023-05534-2 (PMC10654706; doi:10.1038/s42003-023-05534-2)
Supplement: Supplementary file 3 — Description of Additional Supplementary Files [file 42003_2023_5534_MOESM3_ESM.pdf]

Description of Additional Supplementary Files

File name: Supplementary Data 1
Description: Source data supporting the high-throughput conjugation screens and manual validations.

File name: Supplementary Data 2
Description: Source data supporting the heat map and all other manual conjugation assays.
